# AFU PRINCIPLES IN ACTION: ENGAGING STUDENTS THROUGH HANDS-ON AGE-RELATED ACTIVITIES

**DOI:** 10.1093/geroni/igz038.3066

**Published:** 2019-11-08

**Authors:** Cassandra Barragan, Stephanie Wladkowski

**Affiliations:** Eastern Michigan University, Ypsilanti, Michigan, United States

## Abstract

The Age-Friendly University (AFU) Initiative is a global network of universities working to embrace and promote the growing population of older adults (OAs) on campuses. Integrating inter-generational learning is a proven benefit to share knowledge (Gerpott, Lehmann-Willenbrock, & Velopel, 2017) and to mutually benefit both older and more traditional learners (Pstross, Corrigan, Knopf, et al., 2017). To thoughtfully develop AFU initiatives on their campus, one midwestern university created an educational activity for students to better understand the needs of OAs. This presentation will cover results of this activity and offer suggestions for aging-focused learning activities. In winter 2019, 23 undergraduate students from 5 disciplines participated in a guided sensory activity with 5 Masters in Social Work (MSW) students that simulated impaired vision, hearing, and dexterity. Afterwards, using the AARP walking audit, they walked campus to understand challenges those with limitations might face. Students then completed a guided reflection and thought of ways to advocate for anyone with physical challenges, both off and on campus. This activity resulted in several successful learning outcomes and provided concrete experiences, establishing grounds to think about advocacy in a practical way. First, the undergraduate students presented their experiences at a campus-wide activism and advocacy event. They aimed to 1) increase awareness of the challenges those with visual and physical challenges and 2) promote the AFU initiatives. MSW students further analyzed their experience from a policy perspective and presented to the AFU steering committee with recommendations to influence policy in alignment with the AFU principles.

